# Domestication of *Campylobacter jejuni* NCTC 11168

**DOI:** 10.1099/mgen.0.000279

**Published:** 2019-07-16

**Authors:** Ben Pascoe, Lisa K. Williams, Jessica K. Calland, Guillaume Meric, Matthew D. Hitchings, Myles Dyer, Joseph Ryder, Sophie Shaw, Bruno S. Lopes, Cosmin Chintoan-Uta, Elaine Allan, Ana Vidal, Catherine Fearnley, Paul Everest, Justin A. Pachebat, Tristan A. Cogan, Mark P. Stevens, Thomas J. Humphrey, Thomas S. Wilkinson, Alison J. Cody, Frances M. Colles, Keith A. Jolley, Martin C. J. Maiden, Norval Strachan, Bruce M. Pearson, Dennis Linton, Brendan W. Wren, Julian Parkhill, David J. Kelly, Arnoud H. M. van Vliet, Ken J. Forbes, Samuel K. Sheppard

**Affiliations:** ^1^ The Milner Centre for Evolution, University of Bath, Claverton Down, Bath, UK; ^2^ MRC CLIMB Consortium, Bath, UK; ^3^ Swansea University Medical School, Swansea University, Singleton Park, Swansea, UK; ^4^ Cambridge Baker Systems Genomics Initiative, Baker Heart and Diabetes Institute, 75 Commercial Rd, Melbourne 3004, Victoria, Australia; ^5^ Department of Infectious Diseases, Central Clinical School, Monash University, Melbourne, Victoria 3004, Australia; ^6^ University of Aberdeen, Aberdeen, UK; ^7^ Roslin Institute, Edinburgh, UK; ^8^ UCL Eastman Dental Institute, University College of London, London, UK; ^9^ Animal and Plant Health Agency, Weybridge, Surrey, UK; ^10^ University of Glasgow, Glasgow, UK; ^11^ University of Aberystwyth, Aberystwyth, UK; ^12^ School of Clinical Veterinary Science, Langford, Bristol, UK; ^13^ Department of Zoology, University of Oxford, Oxford, UK; ^14^ NIHR Health Protections Research Unit in Gastrointestinal Infections, University of Oxford, Oxford, UK; ^15^ Quadram Institute Bioscience, Norwich, UK; ^16^ University of Manchester, Manchester, UK; ^17^ London School of Hygiene and Tropical Medicine, London, UK; ^18^ Wellcome Sanger Institute, Wellcome Genome Campus, Hinxton, Cambridge, UK; ^19^ Department of Molecular Biology and Biotechnology, The University of Sheffield, Sheffield, UK; ^20^ School of Veterinary Medicine, University of Surrey, Surrey, UK; ^‡^​ Present address: Antimicrobial Resistance Policy and Surveillance Team, Veterinary Medicines Directorate, Department for Environment, Food and Rural Affairs (Defra), Surrey, UK

**Keywords:** *Campylobacter*, genomics, microbial evolution, culture collections

## Abstract

Reference and type strains of well-known bacteria have been a cornerstone of microbiology research for decades. The sharing of well-characterized isolates among laboratories has run in parallel with research efforts and enhanced the reproducibility of experiments, leading to a wealth of knowledge about trait variation in different species and the underlying genetics. *Campylobacter jejuni* strain NCTC 11168, deposited at the National Collection of Type Cultures in 1977, has been adopted widely as a reference strain by researchers worldwide and was the first *Campylobacter* for which the complete genome was published (in 2000). In this study, we collected 23 *C *. *jejuni* NCTC 11168 reference isolates from laboratories across the UK and compared variation in simple laboratory phenotypes with genetic variation in sequenced genomes. Putatively identical isolates, identified previously to have aberrant phenotypes, varied by up to 281 SNPs (in 15 genes) compared to the most recent reference strain. Isolates also display considerable phenotype variation in motility, morphology, growth at 37 °C, invasion of chicken and human cell lines, and susceptibility to ampicillin. This study provides evidence of ongoing evolutionary change among *C. jejuni* isolates as they are cultured in different laboratories and highlights the need for careful consideration of genetic variation within laboratory reference strains. This article contains data hosted by Microreact.

## Data Summary

Short read data are archived on the NCBI SRA associated with BioProject accession PRJNA517467 (https://www.ncbi.nlm.nih.gov/bioproject/PRJNA517467).

Long read data are archived on the ENA associated with BioProject accession PRJEB33069 (https://www.ebi.ac.uk/ena/data/search?query=PRJEB33069).

All assembled genomes are also available on figshare (doi: 10.6084/m9.figshare.7849268). Phylogeny is visualized on microreact: https://microreact.org/project/NCTC11168.

**Impact Statement**

In this paper, we comment on the changing role of laboratory reference strains. While the model organism allows basic comparison within and among laboratories, it is important to remember the effect even small differences in isolate genomes can have on the validity and reproducibility of experimental work. We quantify differences in 23 reference *Campylobacter* genomes and compare them with observable differences in common laboratory phenotypes.

## Introduction

The sharing of bacterial reference or type strains among laboratories is a fundamental part of microbiology. This informal and often uncelebrated enterprise has supported academic, health, food and veterinary research worldwide, underpinning microbiology innovation. The history of the exchange and classification of bacterial type strains has incorporated the work of some of the most influential microbiologists [1]. One such strain belongs to the important food-borne pathogen species *Campylobacter jejuni *.

For *C. jejuni,* the publication of a simplified culturing technique and deposition of a reference isolate at the National Collection of Type Cultures (NCTC 11168) in 1977 (by Martin Skirrow) marked the end of the first century of research into this organism [2]. The first description of an organism likely to be *Campylobacter* was made in Naples in 1884. Theodor Escherich observed spiral bacteria in stool specimens from patients with diarrhoeal disease but he was unable to culture them [3, 4]. Successful isolation of *Bacterium coli commune* (now *Escherichia coli *) from his young dysenteric patients helped pioneer bacterial genetics and lay the foundations of modern microbiology [1, 5]. However, throughout his career, Escherich continued to identify ‘*spirilla*’ in cases of cholera-like and dysenteric disease. It is likely that the microorganisms he described were *Campylobacter* with their typical spiral morphology and association with enteritis [4, 6].

Early in the 20th century researchers investigating veterinary cases of fetal abortion and winter dysentery in cattle [7] described several species that would later become part of the genus *Campylobacter *, including *Vibrio jejuni* [8], *V. fetus* [9], *V. fetus venerealis* and *V. fetus intestinalis* [10]. Isolation techniques that permitted the growth of *Campylobacter* from human faeces drew attention to its importance as a human pathogen [11–13]. The genus name *Campylobacter* (meaning curved rod) was proposed by Sebald and Véron in 1963 and subsequently verified in 1973 with the broader acceptance of *Campylobacter* species as human pathogens [14, 15]. Skirrow’s more convenient culturing technique and the availability of a model reference strain sparked renewed interest in *Campylobacter* research later in the 20th century [16, 17]. Model strains allowed for comparison of experiments within laboratories and isolates were passed among laboratories across the world [18–23]. When the *C. jejuni* NCTC 11168 genome was sequenced in 2000 [24] this type strain was cemented as an important reference strain for *Campylobacter* research. Additional detail was added to the *C. jejuni* genome following its re-annotation (accession: AL11168.1), including revised coding sequence (CDS) identification incorporating potential for phase variation [25–29].

Today, many aspects of the biology of this organism are well characterized. Identification of genomic regions primed for post-translational modification, in particular decoration of surface proteins with glycans [30], pseudaminic acid [31–33] and legionaminic acid [34], have improved understanding of the mechanisms of ganglioside mimicry [35], epithelial cell invasion, host immune-evasion, colonization [36, 37] and development of neurological sequelae such as Guillain-Barré syndrome [38]. Furthermore, insights into virulence traits including strategies to sequester the iron required for infection were detailed using NCTC 11168 [39–41]. Vaccine targets have been identified [42–44] and the mechanisms of core metabolic processes [45, 46], biofilm production [47–51], capsule production [52] and resistance to oxidative stress have been elucidated [53, 54]. Accidental passage through a laboratory worker also identified putative human host adaptations *in vivo* [55].

Since 1977 the NCTC 11168 strain has been an important part of efforts to better understand this pervasive pathogen. However, there are limitations to the use of type strains, the most obvious being that bacteria display considerable variation within species. For example, in *C. jejuni *, some strains cause a significant amount of disease in humans while others do not – owing, in part, to their inability to survive the passage from reservoir host through the food production chain to contaminate human food [56]. This kind of phenotypic variation among strains is well documented in many species and is a central reason for the growing emphasis on population genomics when trying to understand the ecology and evolution of bacteria [57]. A second, more inconspicuous limitation on the use of type strains shared among laboratories is that they might not all be the same. Strains are not *sensu stricto* clones and may display low levels of genetic variation. Clearly, when frozen there is little opportunity for genome evolution to occur [58]. However, whenever there is growth, for example in the process of subculturing isolates, there is an opportunity for genetic variability to be generated within the population. This may be important for interpreting research findings in different groups as even single SNPs can potentially have an impact on phenotype, for example in antimicrobial resistance [59] or host tropism [60]. The aim of the present study was to investigate if, over time, multiple passages under potentially different growth conditions in different laboratories have introduced genotypic and phenotypic variation into a collection of NCTC 11168 *C *. *jejuni *.

## Methods

### Isolates and genome sequencing

Twenty-three laboratory reference *C. jejuni* NCTC 11168 isolates from around the UK were collected and (re)sequenced. The year in which the laboratory received the isolate is noted along with its known heritage (Table 1). DNA was extracted using the QIAamp DNA Mini Kit (Qiagen), according to the manufacturer’s instructions and quantified using a Nanodrop spectrophotometer. Genome sequencing was performed on an Illumina MiSeq sequencer using the Nextera XT Library Preparation Kit. Libraries were sequenced using a 2× 300 bp paired end v3 reagent kit (Illumina). Short read paired-end data were trimmed using trimmomatic (version 0.35; paired-end mode) and assembled using the *de novo* assembly software, SPAdes (version 3.8.0; using the *careful* command). The average number of contigs in the resulting assemblies was 19.7 (range: 13–36) for an average total assembled sequence size of 1 629 408 bp (range: 1 612 402–1 694 909 bp). The average N50 contig length was 173 674 bp (range: 100 444–271 714 bp) (Table S1, available in the online version of this article).

**Table 1.** Summary of genome differences in 23 NCTC 11168 isolates

Isolate	ID	Source laboratory	Variant/comment	Aberrant phenotype*	Genome size (bp)	Total substitutions (snippy)	Genes with substitutions (BIGS)	Number of recombination blocks (Gubbins)	SNPs in recombination (Gubbins)
1	5920	Aberystwyth	Primary lab strain		1 626 801	8	5	0	0
2	5921	Aberdeen	Primary lab strain		1 626 067	11	6	0	0
3	5922	Bristol	Non-motile	✓	1 634 599	26	17	0	0
4	5923	Bristol	Increased motility	✓	1 626 519	22	15	0	0
5	5925	Glasgow	Increased motility	✓	1 625 874	11	7	0	0
6	5926	Glasgow	Original strain		1 625 293	9	9	0	0
7	5927	Glasgow	Sequenced variant		1 626 367	10	8	0	0
8	5928	Norwich	Primary lab strain		1 626 763	32	23	0	0
9	5929	Norwich	Increased motility	✓	1 625 378	15	11	0	0
10	5930	London		1 624 738	14	8	0	0
11	5931	London	Increased motility	✓	1 641 300	13	11	0	0
12	5932	Manchester	Increased motility	✓	1 628 343	12	9	0	0
13	5933	Swansea	Recently purchased		1 694 909	11	6	0	0
14	5934	Oxford	Primary lab strain		1 625 944	11	6	0	0
15	5935	Sheffield	Primary lab strain		1 625 814	59	44	0	0
16	5936	Sheffield	Increased motility	✓	1 626 210	18	16	0	0
17	5937	Sheffield	WT-2000		1 612 402	281	78	4	283
18	5938	Sheffield	WT-2010 (subcultured from WT-2000)		1 625 308	14	11	0	0
19	5939	London	Increased motility	✓	1 625 123	11	10	0	0
20	5940	London	[Genome previously sequenced]		1 625 478
21	5941	Surrey	Primary lab strain		1 625 755	15	9	0	0
22	5942	Surrey	–		1 624 913	17	11	0	0
23	5943	Edinburgh	–		1 626 490	12	9	0	0
Reference	AL11168.1	NCTC	Original sequenced isolate		1 641 481	0	0

*Aberrant phenotypes observed include differences in motility, growth and invasiveness.

### Population structure and phylogenies

Sequence alignments and genome content comparison analyses using blast were performed gene-by-gene, as implemented in the BIGSdb platform [61, 62] as described in previous *Campylobacter* studies [63–66]. A gene was considered present in a given genome when its sequence aligned to an NCTC 11168 locus with more than 70 % sequence identity over at least 50 % of sequence length using blast (File S1, available in the online version of this article) [67]. Genomes were aligned by concatenating single-gene alignments using mafft [68]. For context, collected NCTC 11168 isolates were augmented with 83 previously published genomes representing the known genetic diversity in *C. jejuni* (Table S2). Genes present in 90 % or more of the isolate genomes were aligned (1 359 883 bp; File S2) and a maximum-likelihood phylogeny was reconstructed in FastTree (version 2.1.10; with the generalized time reversible substitution model) [69]. A second alignment of just the collected NCTC 11168 strains was made (1 555 326 bp; File S3) to build an additional maximum-likelihood tree, which was used as input for ClonalFrame-ML to mask putative recombination sites (version 1.11–3) [70] and visualized in microreact: https://microreact.org/project/NCTC11168 [71].

### Estimating genome variation

Sequence reads were compared to the completed NCTC 11168 reference genome (AL11168.1) using snippy (version 3.2dev; File S4) [72] to estimate nucleotide differences between our laboratory reference isolates and the originally sequenced genome. Assembled genomes were annotated with prokka (version 1.13) [73] and the number of polymorphisms introduced by mutation and recombination was inferred using Gubbins (version 2.3.1) [71] for each isolate (per branch; File S5). All high-performance computation was performed on mrc climb in a conda environment [74, 75].

### Phenotype testing

Isolates were recovered from frozen storage on Columbia blood agar (E and O Labs) and incubated under microaerobic conditions at 37 °C and subcultured in Mueller-Hinton broth (Oxoid) and grown microaerobically overnight at 37 °C.

### Bacterial growth assays

Broth cultures were standardized to an OD_600_ of 0.05. For growth curves at 37 and 42 °C, 20 µl of the standardized broth culture was added to 180 µl of Mueller-Hinton broth in a microtitre plate. Optical densities were measured at hourly intervals over a period of 48 h using an OMEGA FLUOstar (BMG LabTech) plate reader with an atmospheric environment of 10 % CO_2_ and 3 % O_2_. Growth curve assays were performed in triplicate, with three technical replicates for each biological replicate. Multiple comparisons among isolates at 37 and 42 °C were compared using a one-way ANOVA with a Tukey post-test [76].

### Swarming assays and motility

For each isolate, a 1 ml aliquot of the standardized preculture (OD_600_ 0.05) was transferred to 5 ml of fresh Mueller-Hinton broth and 2 µl was pipetted onto the centre of semi-solid Mueller-Hinton agar [11.5 g Muller Hinton broth, 2.5 g Agar 3 (Oxoid) in 500 ml deionized water] and incubated at 42 °C for 24 h. Variation in isolate swarming was observed on Mueller-Hinton motility plates. Motile isolates spread across the plates and halo diameters were measured after 1 day of incubation. Isolates were grouped into three categories: non-motile isolates did not spread across the plate; isolates with halo diameters up to 1.5 cm were categorized as motile; and those with halos of a diameter above 1.5 cm were designated as hyper-motile [36].

### Invasion assays

A chicken gut epithelial cell line (MM-CHiC clone, 8E11; Micromol) and a human colon epithelial adenocarcinoma cell line (HT-29) were used to assay invasion of *Campylobacter in vivo*. A 24-well plate was seeded with 8E11 cells in assay medium [modified McCoy’s 5A/DMEM/F-12 with l-glutamine (5 mM) and supplemented with 5 % FBS] and incubated at 37 °C in 5 % CO_2_ for between 4 and 7 days. Liquid cultures were standardized by diluting with Mueller-Hinton broth to between 0.030 and 0.080. Aliquots of 200 µl from each isolate were deposited into a 96-well plate and diluted serially. The original stock and dilutions were spread onto Columbia horse blood agar and incubated for 24 h microaerobically at 42 °C. Once the cells had reached confluent growth, the medium was removed and the monolayer was washed three times with warm PBS. An aliquot of 1 ml pre-warmed antibiotic-free supplemented Dulbecco's modified Eagle medium (DMEM) was added to each well and inoculated with 100 µl 1×10^7^ c.f.u. Following incubation in 5 % CO_2_ at 37 °C for 4 h, the cells were washed twice with 2 ml PBS supplemented with 4 µl (100 µl ml^−1^) gentamicin and incubated for a further 1.5 h. Cells were washed three times with PBS and an aliquot of 1 ml of warmed TrypLE (Gibco) was added to each well and incubated at 37 °C for 10 min. The lysed monolayer solution was diluted serially and spread onto Columbia horse blood agar in duplicate. Plates were incubated overnight at 42 °C in a microaerobic environment and enumerated pre- and post-invasion to calculate the percentage of invaded inoculum. Assays with human HT-29 cells were performed with McCoys growth media. Invasion assays were performed in triplicate and analysed using unpaired *t*-tests with Welch’s correction.

## Results and Discussion

### Not all reference strains are equal

**Fig. 1.**
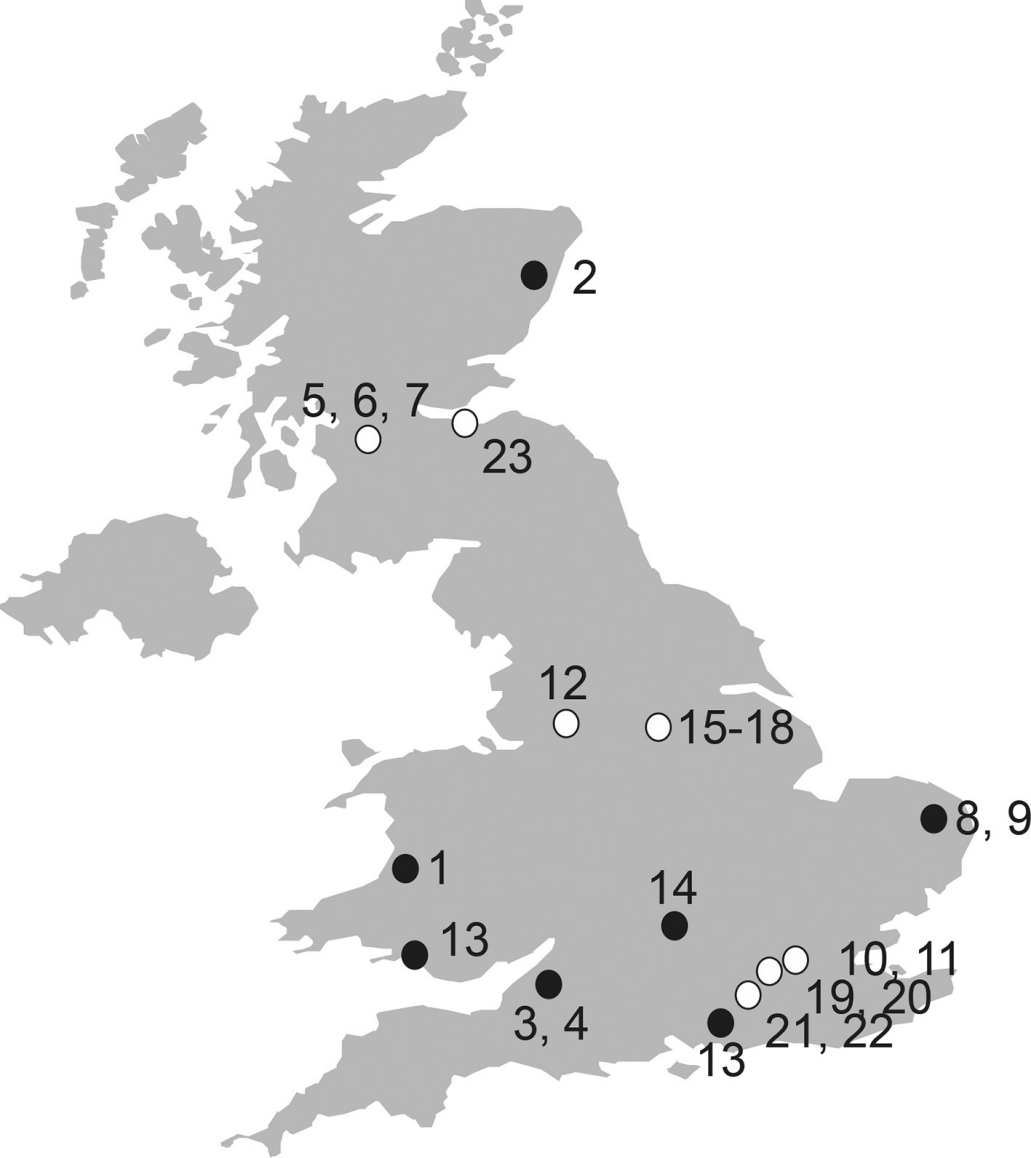
The location of laboratories contributing *C. jejuni* NCTC 11168 isolates. The most recent NCTC 11168 isolate was obtained by Swansea (isolate 13) in 2016 from the NCTC collection. Other isolates obtained directly from the NCTC collection are coloured black, while isolates obtained via a second laboratory are coloured white.

**Fig. 2.**
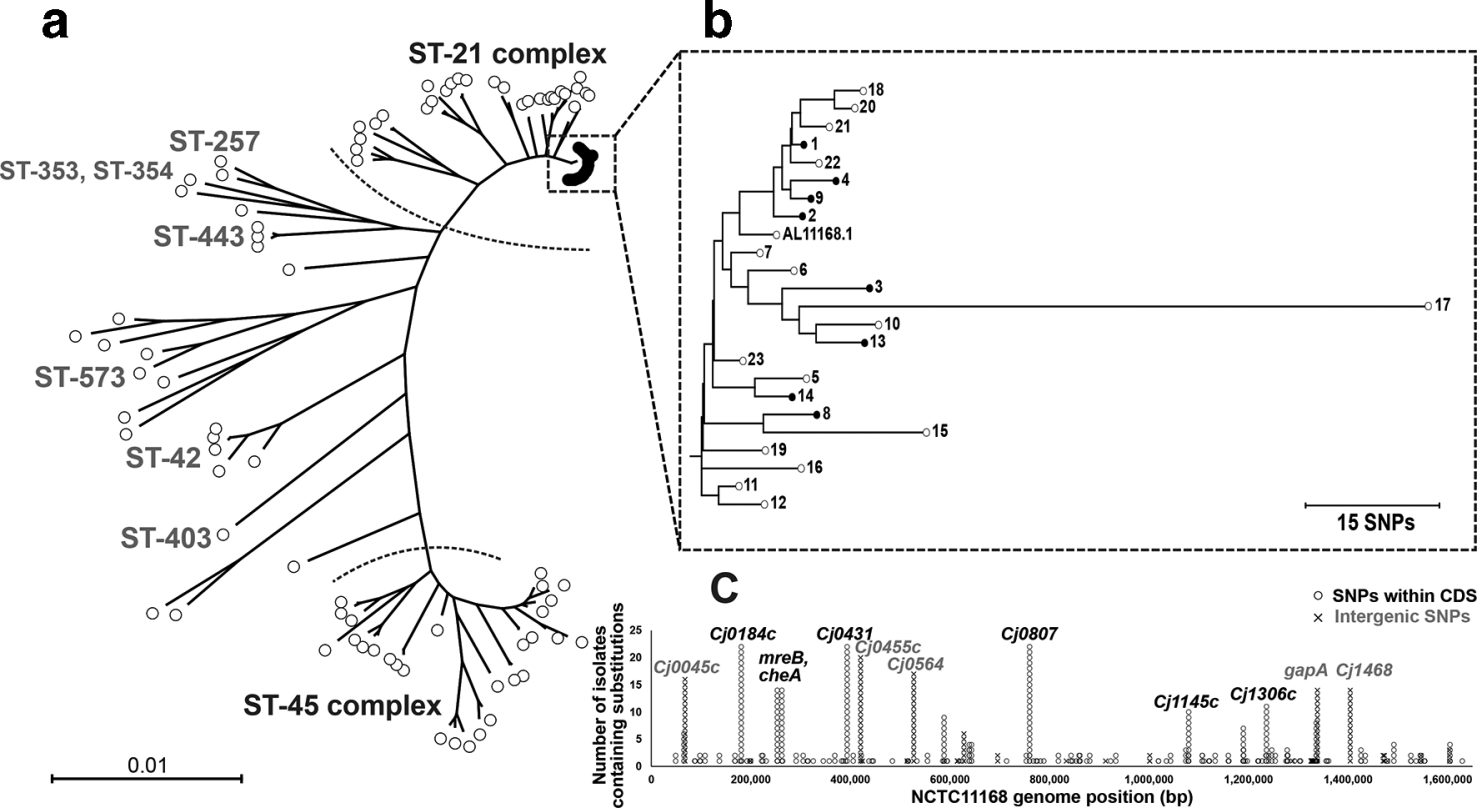
Genetic variation among *C. jejuni* NCTC 11168 genomes. (a) NCTC 11168 isolates were contextualized with 83 previously published genomes representing the known genetic diversity in *C. jejuni* (total of 106 isolates). Genes present in 90 % or more of the isolate genomes were aligned (1 359 883 bp) and a maximum-likelihood phylogeny was reconstructed in FastTree2 with the generalized time reversible substitution model. Bar, genetic distance of 0.01. (b) Recombination was masked using ClonalFrame-ML to produce an alignment of the NCTC 11168 isolates only (*n*=23; 1 555 326 bp). Bar, 15 nt substitutions. (c) The position of all nucleotide substitutions identified using snippy were mapped against the original NCTC 11168 genome (AL11168.1). SNPs found within coding regions (CDS) are represented with circles and SNPs located in intergenic regions are represented with an X. Gene names are given where variation was observed in 10 or more of the isolates.

Since its deposition at the NCTC there have been two main dissemination hubs of NCTC 11168. Ten of the 23 isolates we collected were obtained by contributing laboratories directly from the NCTC collection, while 13 isolates had come via another laboratory (Fig. 1). DNA was extracted from each isolate and sequenced, and the genome was assembled (Table S1). All 23 isolates clustered closely in the host-generalist ST-21 lineage when compared on a maximum-likelihood phylogenetic tree (Fig. 2a). This suggests that despite some phenotypic heterogeneity, all isolates derived were from a recent common ancestor and no strains were misidentified during passage. Micro-evolutionary differences among closely related NCTC 11168 isolates were observed on a recombination-free phylogeny constructed using ClonalFrameML (Fig. 2b). Genomes were compared to the original NCTC 11168 genome and as many as 281 SNP differences were observed (up to 15 genes in isolate 17) among collected laboratory strains and the reference (Fig. 2c; Table 1). However, in 21 of 23 isolates (91%) there were 32 or fewer SNP differences compared to the reference (Table 1). There was an average of 29 SNP differences between the laboratory strains and the reference, and the fewest SNPs in any comparison was eight SNP differences (in five genes in isolate 1).

Under ideal storage conditions one might not expect to see any evidence of recent recombination in the laboratory reference strains. Nevertheless, we estimated the number of mutations and recombination events using Gubbins. In total, 436 of the 632 SNPs (69%) we identified were found within protein coding regions, of which 83 were synonymous mutations (19 %; File S5). The only isolate where we inferred any recombination was isolate 17, which has acquired four recombination blocks comprising a total of 14 816 bp, incorporating 283 SNPs. In comparison, 29 SNPs were identified outside these recombination blocks (as a result of mutation), corresponding to a ratio at which recombination introduces nucleotide changes, relative to mutation (*r*/*m)* of 9.76 (File S5). This isolate also lost a block of 15 genes (*Cj1319-1333*; File S1), which includes a *maf*-family gene (*maf3/Cj1334*) involved in post-translational modification of flagellins. Also missing were the *neuC2/Cj1328*, *neuB2/Cj1327*, *ptmA/Cj1332* and *ptmB/Cj1331* genes involved in the addition of pseuaminic/legionaminic acid to *C. jejuni* flagellins [32, 77, 78]. A knockout mutant of the final gene in this block, *Cj1333*, demonstrated compromised agglutination and reduced invasion (in INT-407 cells) [78]. This region of the *C. jejuni* genome is prone to recombination and has shown a high level of diversity and is often implicated in bacterial virulence [34, 35, 37, 79–82]. Isolate 17 was hyper-motile and also among the most invasive isolates when tested against chicken cell lines, but invaded human cell lines poorly (Table 2).

**Table 2.** Summary of phenotype differences in 23 NCTC 11168 isolates

Isolate	ID	Source laboratory	Variant/comment	Aberrant phenotype	Observed motility	Maximum growth at 37 °C (OD_600_)	Maximum growth at 42 °C (OD_600_)	% invaded (HT-29 cell line)	% invaded (chicken cell line)	Ampicillin MIC (µg ml^−1^)	blaOXA-61
1	5920	Aberystwyth	Primary lab strain		–	–	–	–	–	–	1
2	5921	Aberdeen	Primary lab strain		Non-motile	0.024	0.331	3	2	2	1
3	5922	Bristol	Non-motile	✓	Non-motile	0.032	0.364	1	1	8	1
4	5923	Bristol	Increased motility	✓	Motile	0.040	0.216	2	5	2	1
5	5925	Glasgow	Increased motility	✓	Motile	0.042	0.286	2	2	4	1
6	5926	Glasgow	Original strain		Motile	0.049	0.270	1	2	0.015	1
7	5927	Glasgow	Sequenced variant		Motile	0.039	0.277	2	2	2	1
8	5928	Norwich	Primary lab strain		Motile	0.017	0.236	1	1	8	1
9	5929	Norwich	Increased motility	✓	Motile	0.081	0.599	1	3	0.015	1
10	5930	London	–		Hyper-motile	0.021	0.338	3	2	4	1
11	5931	London	Increased motility	✓	Hyper-motile	0.034	0.135	3	2	2	1
12	5932	Manchester	Increased motility	✓	Motile	0.028	0.321	8	1	4	1
13	5933	Swansea	Recently purchased		Non-motile	0.119	0.250	1	3	1	1
14	5934	Oxford	Primary lab strain		Non-motile	0.042	0.323	1	4	1	1
15	5935	Sheffield	Primary lab strain		Motile	0.001	0.205	4	6	0.015	1
16	5936	Sheffield	Increased motility	✓	Motile	0.034	0.077	2	5	4	1
17	5937	Sheffield	WT-2000		Hyper-motile	0.023	0.304	1	3	4	1
18	5938	Sheffield	WT-2010 (subcultured from WT-2000)		Motile	0.018	0.289	0	2	0.015	1
19	5939	London	Hyper-motile	✓	Motile	0.086	0.172	0	8	1	1
20	5940	London	[Genome previously sequenced]		–	–	–	–	–	–	0
21	5941	Surrey	Primary lab strain		Non-motile	0.046	0.236	1	0	1	1
22	5942	Surrey	–		Motile	0.084	0.226	1	1	0.015	1
23	5943	Edinburgh	–		Motile	0.095	0.245	1	2	2	1
Reference	AL11168.1	NCTC	Original sequenced isolate		–	–	–	–	–	–	0

Isolate motility was tested *in vitro* [83] and phenotypic variation was observed among NCTC 11168 isolates (Table 2). Since its original dissemination, motile, non-motile and hyper-motile variants have been reported [25, 28, 84]. Despite previous observations describing increased motility, only three strains were categorized as hyper-motile in our assays (swarming >1.5 cm), all of which had been passed between at least two laboratories before entering our collection. Only 50 % of the isolates received by laboratories directly from the NCTC collection were motile (Table 2). Changes in motility can be a result of differences in the *flaA* and *flaB* genes resulting in attenuated flagella assembly [36]. However, we did not identify any non-synonymous mutations within the *flaA* or *flaB* genes. A shared frameshift mutation was identified in two hyper-motile isolates (11 and 16) within the core motor protein, *fliR* [85–87]. Isolate motility is also influenced by phase-variable gene expression as a result of upstream homopolymeric repeat regions [24, 88, 89]. Three motility-associated genes (*maf1/Cj1348*, *maf4/Cj1335* and *maf7/Cj1342c*) were among 31 phase-variable regions recently identified in NCTC 11168 [90] and were among SNPs we identified in non-coding intergenic regions (196 of 632; 31 %; File S4). Twelve genes (*Cj0045c*, *Cj0184*, *mreB*, *cheA*, *Cj0431*, *Cj0455c*, *Cj0564*, *Cj0807*, *Cj1145c*, *Cj1306c*, *gapA* and *Cj1468*) contained nucleotide polymorphisms in 10 or more NCTC 11168 isolates, of which five have been shown to be subject to phase variation (*Cj0045c*, *Cj0455c*, *Cj0564*, *gapA* and *Cj1468*; Fig. 2c) [89]. Growth of motile bacteria in culture media can result in loss of motility as flagella construction is energetically expensive [91, 92]. In batch culture, rapid growth is prioritized and loss of flagella can be advantageous [93, 94].

**Fig. 3.**
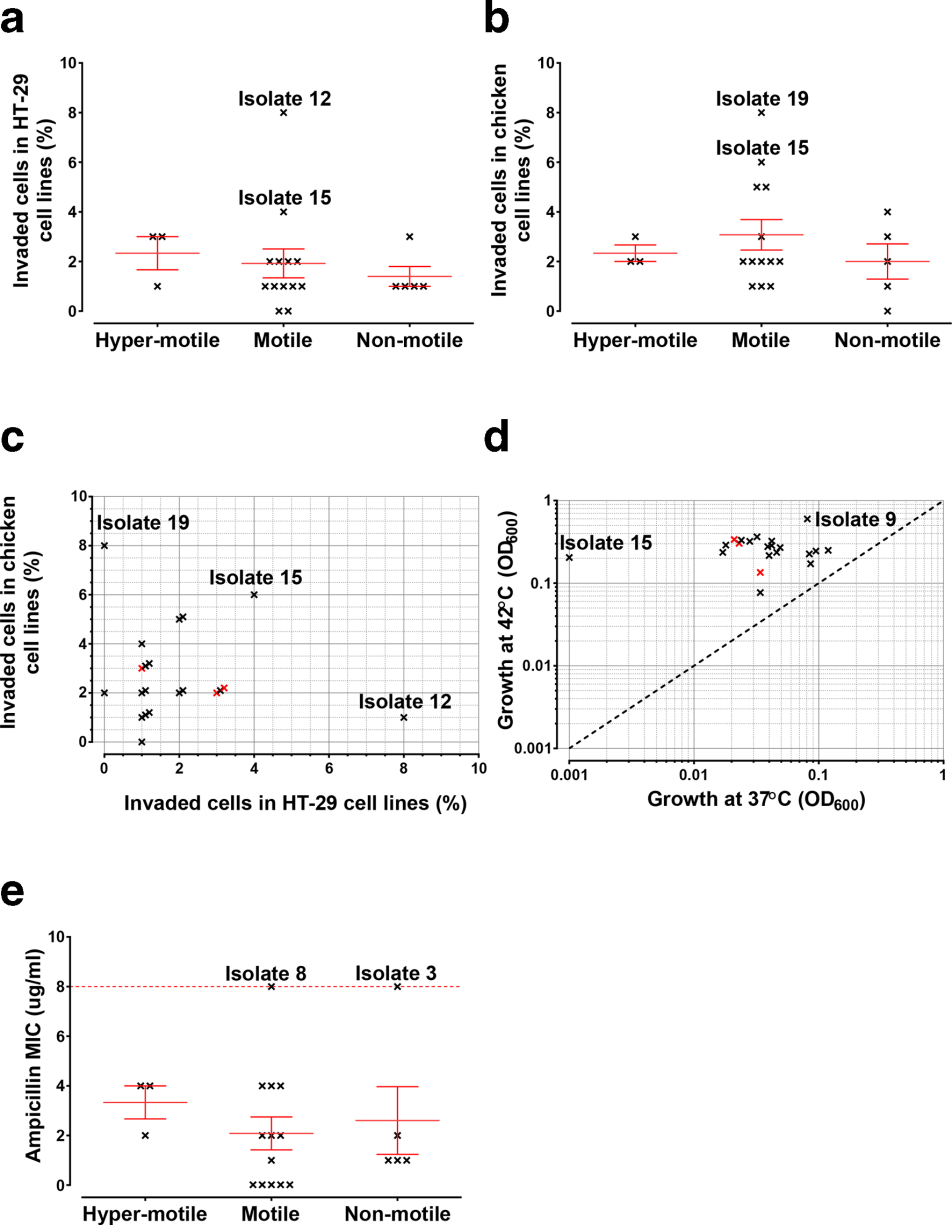
Phenotypc variation among *C. jejuni* NCTC11168 genomes. Invasion assays were carried out for strains categorized by motility phenotypes in (a) human HT-29 and (b) chicken cell lines. For each isolate the percentage of invaded original inocula is plotted with a mean line and bars representing sem. Comparisons were made between (c) invasiveness in these cell lines and (d) maximum growth at different temperatures, with hyper-motile isolates coloured red. The minimum inhibitory concentration (MIC) of ampicillin was determined for isolates grouped by motility (e).

Adequate flagella construction is an important virulence factor because, in addition to motility, flagella also contribute to invasion and secretion [95, 96], without which colonization is impaired [28]. The ability of isolates to invade human and chicken intestinal epithelial cell lines was tested *in vitro* by a gentamicin protection assay (Fig. 3a, b). Fourteen of 21 isolates tested invaded the 8E11 chicken cell line more effectively compared to the human HT-29 cell line (Fig. 3c). On average, motile (*n*=13, 2 and 3 % of the original inoculum invaded chicken and human cell lines, respectively) and hyper-motile isolates (*n*=3; 2 % of the original inoculum invaded chicken and human cell lines) invaded both cell lines in greater numbers than non-motile isolates (*n*=5; 1 and 2 % of the original inoculum invaded chicken and human cell lines, respectively; Table 2; Fig. 3a, b). Several genes containing SNPs in multiple isolates have been shown previously to contribute to increased invasion and virulence, including *mreB* (*n*=14), *cheA* (*n*=14), *Cj0431* (*n*=22), *Cj0455* (*n*=20), *Cj0807* (*n*=22) and *Cj1145* (*n*=10) [55, 81, 97]. Isolate growth was tested at 37 and 42 °C, with all growing to a higher optical density at avian body temperature (42 °C) (Fig. 3d). Isolate 15 grew particularly poorly at 37 °C. We identified the OXA-61 gene in the majority of isolates, but only two were resistant to ampicillin, according to CLSI guidelines (isolates 3 and 8; Table 2; Fig. 3e) [98]. No SNP changes were observed in *cmeABC* genes in any isolate (File S1).

### The role of model strains in an age of population genomics

In most cases (21 of 23 isolates; 91%) we observed fewer than 32 SNPs between the laboratory isolate and the type strain deposited in the NCTC archive. However, even these minor changes are associated with observable phenotypic differences (motility and invasion as seen here). This could be seen as a challenge to the reproducibility of experiments in different laboratories that use ostensibly identical strains [55, 97]. It is accepted among microbiologists that there is potential for variation among type strains that may display considerable genome plasticity, such as in *Helicobacter pylori* [99]. Consistent with this, variants of *C. jejuni* NCTC 11168 are defined as motile/non-motile, colonizer/non-colonizer for use in specific experiments.

Technical advances in high-throughput genome sequencing and analysis methods continue to improve understanding of *C. jejuni* from bottom-up studies that test the function of specific genes or operons, often with insertion or deletion mutants [55, 97], to top-down comparative genomic approaches in which isolates are clustered by phenotype and associated genomic variations are identified in large genome collections [50, 64, 100]. Early genome typing using DNA microarrays hinted at the level of diversity among *C. jejuni* isolates [27, 101], and comparisons of large isolate genome collections are now linking strain variation to differences in ecology [65, 102–105], epidemiology and evolution [63, 100, 106–110]. Advances in sequencing technology are helping us to study genome variation in greater depth, and long read sequencing of isolate 2 identified large inversions (>90 000 bp) compared to the original finished genome (Table S1).

In conclusion, the genotypic and phenotypic differences among NCTC 11168 strains in this study, probably as a result of evolution during repeated passages, emphasizes the need for laboratories to maintain isolate collections with detailed records and good culture practices. This essentially reaffirms the work of microbiology pioneers who developed practices to minimize variation between strains and laboratories. However, in the genomics era, it may also be prudent to sequence strains more routinely, particularly as the costs continue to decline. While the interpretation of experiments using reference type strains may be adapting to more detailed genomic data and improved understanding of genome evolution, the strains themselves remain an essential resource in microbiology. The perceived power of large-scale comparative genomics and statistical genetics studies typically lies in the ability to identify genes or genetic variation that confers putative functional differences to the bacterium. Confirming these associated gene functions [56] requires traditional microbiology based upon a detailed understanding of reliable reference type control strains such as NCTC 11168.

## Data bibliography

1. Pascoe B, Williams L. K., Calland J. K., Méric G, Hitchings M. D., Dyer M, Ryder J, Allen E, Vidal A, Fearnley C, Everest P, Linton D, Pachebat J. A., Cogan T. A., Stevens M. P., Wilkinson T. S., Humphrey T. J., Cody A. J., Colles F. M., Jolley K. A., Maiden M. C. J, Forbes K, Strachan N, Kelly D. J., Pearson B. M., Wren B. W., Parkhill J, van Vliet A. H. M., Sheppard S. K. Sequence Read Archive (SRA),BioProject Accession PRJNA517467 (2019).

2. Sheppard S. K., Didelot X, Jolley K. A., Darling A. E., Pascoe B, Méric G, Kelly D. J., Cody A. J., Colles F. M., Strachan N. J., Ogden I. D., Forbes K, French N. P., Carter P, Miller W. G., McCarthy N. D., Owen R, Litrup E, Egholm M, Affourtit J. P., Bentley S. D., Parkhill J, Maiden M. C. J., Falush D. Sequence Read Archive (SRA), BioProject Accession PRJNA177352 (2013).

3. Sheppard S. K., Didelot X, Méric G, Torralbo A, Jolley K. A., Kelly D. J., Bentley S. D., Maiden M. C. J., Parkhill J, Falush D. European Nucleotide Archive (ENA),Study Accession ERP000129 (2013).

4. Pearson B. M., Gaskin D. J., Segers R. P., Wells J. M., Nuijten P. J., van Vliet A. H. GenBank sequence: CP000814.1 (2007).

5. Fouts D, Nelson K, Sebastian Y. GenBank sequence: CP000538.1 (2006).

6. Fouts D. E., Mongodin E. F., Mandrell R. E., Miller W. G., Rasko D. A., Ravel J, Brinkac L. M., DeBoy R. T., Parker C. T., Daugherty S. C., Dodson R. J., Durkin A. S., Madupu R, Sullivan S. A., Shetty J. U., Ayodeji M. A., Shvartsbeyn A, Schatz M. C., Badger J. H., Fraser C. M., Nelson K. E. GenBank sequence: CP000025.1 (2004).

7. Gundogdu O, Bentley S. D., Holden M. T., Parkhill J, Dorrell N, Wren B. W. GenBank sequence: AL111168.1 (2006).

8. The University of Aberdeen. European nucleotide archive (ENA), BioProject accession PRJEB33069 (2019)

## Supplementary Data

Supplementary File 1

Supplementary File 2
