# Supplementary material for: Domestication of Campylobacter jejuni NCTC 11168
Source: Microb Genom. 2019 Jul 16;5(7):e000279. doi: 10.1099/mgen.0.000279 (PMC6700657; doi:10.1099/mgen.0.000279)
Supplement: Supplementary File 1 [file mgen-5-279-s001.pdf]

Isolate	ID	Source laboratory	Variant / comment	Original source	Archived	Number of contigs	Genome size (bp)	N50 contig length (L50)	SRA accession	GenBank accession	BioProject
1	5920	Aberystwyth	Primary lab strain	NCTC	2015	18	1,626,801	174,210	SRR8731760	VCLY00000000	PRJNA517467
2	5921	Aberdeen	Primary lab strain	NCTC	2002	16	1,626,067	174,212	SRR8731759	VCLX00000000	PRJNA517467
3	5922	Bristol	Non-motile	NCTC	2002	36	1,634,599	189,487	SRR8731758	VCLW00000000	PRJNA517467
4	5923	Bristol	Hyper-motile	NCTC	2005	18	1,626,519	154,057	SRR8731757	VCLV00000000	PRJNA517467
5	5925	Glasgow	Hyper-motile	London	2000	22	1,625,874	173,183	SRR8731764	VCLU00000000	PRJNA517467
6	5926	Glasgow	Original strain	NCTC (via Martin Skirrow)	2000	16	1,625,293	154,057	SRR8731763	VCLT00000000	PRJNA517467
7	5927	Glasgow	Sequenced variant	London	2000	23	1,626,367	173,039	SRR8731762	VCLS00000000	PRJNA517467
8	5928	Norwich	Primary lab strain	NCTC	2004	19	1,626,763	188,103	SRR8731761	VCLR00000000	PRJNA517467
9	5929	Norwich	Hyper-motile	NCTC	2004	31	1,625,378	100,444	SRR8731766	VCLQ00000000	PRJNA517467
10	5930	London	--	London	2000	35	1,624,738	108,757	SRR8731765	VCLP00000000	PRJNA517467
11	5931	London	Hyper-motile	London	2000	20	1,641,300	188,163	SRR8731768	VCLO00000000	PRJNA517467
12	5932	Manchester	Hyper-motile	London	2003	25	1,628,343	188,003	SRR8731767	VCLN00000000	PRJNA517467
13	5933	Swansea	Recently purchased	NCTC	2016	16	1,694,909	153,963	SRR9165868	VCLM00000000	PRJNA517467
14	5934	Oxford	Primary lab strain	NCTC	2014	19	1,625,944	122,646	SRR8731770	VCLL00000000	PRJNA517467
15	5935	Sheffield	Primary lab strain	London	2015	14	1,625,814	189,487	SRR8731769	VCLK00000000	PRJNA517467
16	5936	Sheffield	Hyper-motile	London	2013	14	1,626,210	189,487	SRR8731772	VCLJ00000000	PRJNA517467
17	5937	Sheffield	WT-2000	London (via Birmingham)	2000	15	1,612,402	189,478	SRR8731771	VCLI00000000	PRJNA517467
18	5938	Sheffield	WT-2010 (subcultured from WT-2000)	London	2010	13	1,625,308	189,488	SRR8731774	VCLH00000000	PRJNA517467
19	5939	London	Hyper-motile	London	2002	14	1,625,123	189,490	SRR8731773	VCLG00000000	PRJNA517467
20	5940	London	[Genome previously sequenced]	London	2002	18	1,625,478	189,490	NA	VCLF00000000	PRJNA517467
21	5941	Surrey	Primary lab strain	NCTC (via Cambridge)	2000	16	1,625,755	271,714	SRR8731776	VCLE00000000	PRJNA517467
22	5942	Surrey	--	NCTC (via Cambridge)	2000	16	1,624,913	154,057	SRR8731775	VCLD00000000	PRJNA517467
23	5943	Edinburgh	--	London (via Sheffield)	2013	20	1,626,490	189,488	SRR8731777	VCLC00000000	PRJNA517467
LR2	--	Aberdeen	Long read genome	NCTC	2002	tbc	tbc	tbc	tbc	--	tbc
Reference	--	NCTC	Original sequenced isolate	London	2000	1	1,641,481	1,641,481	--	GCA_000009085.1	PRJNA8
