# Supplementary material for: Domestication of Campylobacter jejuni NCTC 11168
Source: Microb Genom. 2019 Jul 16;5(7):e000279. doi: 10.1099/mgen.0.000279 (PMC6700657; doi:10.1099/mgen.0.000279)
Supplement: Supplementary File 2 [file mgen-5-279-s002.pdf]

ID	Isolate	Country	Year	Source	Genome size (bp)	Contigs	ST	Clonal complex	BioProject	Accession
4	CAMP45	UK	2005	chicken	1,596,969	108	45	ST-45	PRJNA177352	ANGO00000000.1
22	CAMP2488	UK	2001	chicken	1,573,736	169	257	ST-257	ERP000129	ERS007807
26	NCTC11828	UK	2007	Lab strain	1,628,115	1	267	ST-283	PRJNA17953	CP000814.1
27	NC 008787	USA	2007	Lab strain	1,616,554	1	604	ST-42	PRJNA224116	CP000538.1
28	RM1221	USA	2002	Lab strain	1,777,831	1	354	ST-354	PRJNA224116	CP000025.1
**29**	**NCTC11168**	**UK**	**2000**	Lab strain	**1,641,481**	**1**	**43**	**ST-21**	**PRJNA57587**	**AL111168.1**
30	CAMP1044	UK	2007	Lab strain	1,613,621	465	--	--	PRJNA177352	ANHK00000000.1
32	CampsClin11	UK	2005	clinical	1,650,105	92	11	ST-45	ERP000129	ERR024478
34	CampsClin262	UK	2005	clinical	1,643,032	91	262	ST-21	ERP000129	ERR024475
36	CampsClin266	UK	2006	clinical	1,695,272	47	266	ST-21	ERP000129	ERR024476
37	CampsClin883	UK	2006	clinical	1,667,560	113	883	ST-21	ERP000129	ERR024477
39	chick2219	UK	2005	chicken	1,616,482	84	2219	ST-45	ERP000129	ERR024426
40	chicka21	UK	2006	chicken	1,726,327	195	21	ST-21	ERP000129	ERR024431
42	cow42	UK	2006	cattle	1,672,737	41	42	ST-42	ERP000129	ERR024430
45	chick594	UK	2006	chicken	1,609,163	72	583	ST-45	ERP000129	ERR024434
47	cow2674	UK	2006	cattle	3,453,740	455	21	ST-21	ERP000129	ERR024436
48	cow206	UK	2006	cattle	1,671,619	62	206	ST-206	ERP000129	ERR024437
49	cow38	UK	2006	cattle	1,663,148	181	38	ST-48	ERP000129	ERR024427
52	cow334	UK	2006	cattle	1,616,567	102	334	ST-45	ERP000129	ERR023263
54	chick267	UK	2005	chicken	1,591,217	241	267	ST-283	ERP000129	ERR023268
55	CampsClin230	UK	2006	clinical	1,625,711	271	230	ST-45	ERP000129	ERR024480
56	cowa45	UK	2006	cattle	1,607,778	62	45	ST-45	ERP000129	ERR023269
57	chick2213	UK	2005	chicken	1,620,325	158	334	ST-45	ERP000129	ERR023270
59	cow518	UK	2006	cattle	1,705,325	60	21	ST-21	ERP000129	ERR024441
60	CampsClin53	UK	2005	clinical	1,658,292	58	53	ST-21	ERP000129	ERR024474
62	cowa21	UK	2006	cattle	1,658,436	100	21	ST-21	ERP000129	ERR023273
63	chickc21	UK	2006	chicken	1,679,349	139	21	ST-21	ERP000129	ERR024447
64	chick25	UK	2006	chicken	1,698,035	113	814	ST-661	ERP000129	ERR024448
65	chick104	UK	2006	chicken	1,761,202	112	104	ST-21	ERP000129	ERR023274
66	chick353	UK	2009	chicken	1,776,210	129	353	ST-353	ERP000129	ERR023264
67	chickb354	UK	2009	chicken	1,688,706	143	354	ST-354	ERP000129	ERR024453
68	chick573	UK	2009	chicken	1,838,022	251	573	ST-573	ERP000129	ERR023265
69	chick2568	UK	2009	chicken	1,821,236	144	2568	ST-661	ERP000129	ERR024456
70	chickc45	UK	2009	chicken	1,595,762	343	45	ST-45	ERP000129	ERR023266
71	chick19	UK	2009	chicken	1,689,713	84	50	ST-21	ERP000129	ERR023276
72	chick50	UK	2009	chicken	1,692,341	63	50	ST-21	ERP000129	ERR023280
73	chick53	UK	2009	chicken	1,651,079	102	53	ST-21	ERP000129	ERR023281
74	chick262	UK	2009	chicken	1,606,379	65	262	ST-21	ERP000129	ERR023282
75	chick266	UK	2009	chicken	1,693,845	76	266	ST-21	ERP000129	ERR023283
77	chick1086	UK	2009	chicken	1,692,435	65	50	ST-21	ERP000129	ERR023285
78	chick1360	UK	2009	chicken	1,693,941	67	50	ST-21	ERP000129	ERR023286
79	chick11	UK	2009	chicken	1,645,238	120	11	ST-45	ERP000129	ERR023287
80	chick137	UK	2009	chicken	1,734,017	73	2030	ST-257	ERP000129	ERR023277
81	chick1003	UK	2009	chicken	1,617,200	106	1003	ST-45	ERP000129	ERR023278
82	chick2048	UK	2009	chicken	1,631,119	163	45	ST-45	ERP000129	ERR024450
84	chick2223	UK	2009	chicken	1,605,483	58	45	ST-45	ERP000129	ERR024452
85	cow3583	UK	2003	cattle	1,654,563	93	3583	ST-42	ERP000129	ERR024442
87	cow273	UK	2003	cattle	1,715,399	62	273	ST-206	ERP000129	ERR024445
88	cow270	UK	2003	cattle	1,716,038	72	270	ST-403	ERP000129	ERR023293
89	cowb21	UK	2003	cattle	1,659,711	130	21	ST-21	ERP000129	ERR023294
90	cowb45	UK	2003	cattle	1,603,131	64	45	ST-45	ERP000129	ERR023295
91	cowc45	UK	2003	cattle	1,602,224	47	45	ST-45	ERP000129	ERR024443
92	cowd45	UK	2003	cattle	1,607,691	98	45	ST-45	ERP000129	ERR024444
94	cow104	UK	2003	cattle	1,762,939	82	104	ST-21	ERP000129	ERR024439
97	cow3201	UK	2003	cattle	1,629,692	114	19	ST-21	ERP000129	ERR023299
99	cow3205	UK	2003	cattle	1,720,953	64	206	ST-206	ERP000129	ERR023290
100	cow137	UK	2003	cattle	1,626,334	192	137	ST-45	ERP000129	ERR023291
102	cow583	UK	2003	cattle	1,607,515	63	583	ST-45	ERP000129	ERR027216
103	cow3207	UK	2003	cattle	1,641,790	70	334	ST-45	ERP000129	ERR027220
104	cow3214	UK	2003	cattle	1,654,338	115	45	ST-45	ERP000129	ERR027221
105	chick354	UK	2004	chicken	1,697,013	75	257	ST-257	ERP000129	ERR027222
106	chick51	UK	2005	chicken	1,714,044	58	51	ST-443	ERP000129	ERR024454
107	chick1079	UK	2004	chicken	1,838,061	273	1079	ST-573	ERP000129	ERR024455
108	chick574	UK	2004	chicken	1,743,461	80	574	ST-574	ERP000129	ERR027223
109	chick814	UK	2004	chicken	1,759,789	152	814	ST-661	ERP000129	ERR027224
110	chickb21	UK	2003	chicken	1,656,261	72	21	ST-21	ERP000129	ERR027225
111	chickb45	UK	2004	chicken	1,649,834	80	45	ST-45	ERP000129	ERR027226
112	chickd45	UK	2004	chicken	1,618,948	55	45	ST-45	ERP000129	ERR024440
113	chick883	UK	2004	chicken	1,665,144	72	883	ST-21	ERP000129	ERR027227
114	chick230	UK	2004	chicken	1,633,592	79	230	ST-45	ERP000129	ERR027217
116	CampsClin21	UK	2005	clinical	1,656,471	65	9092	ST-21	ERP000129	ERR024472
117	OxClina21	UK	2003	clinical	1,697,696	113	21	ST-21	ERP000129	ERR024457
119	OxClina45	UK	2003	clinical	1,621,668	66	45	ST-45	ERP000129	ERR024459
122	starling177	UK	2007	starling	1,582,720	52	177	ST-177	ERP000129	ERR024461
124	starling45	UK	2007	starling	1,603,669	112	45	ST-45	ERP000129	ERR024464
125	starling1020	UK	2007	starling	1,578,916	61	1020	ST-682	ERP000129	ERR024465
126	goose1033	UK	2007	goose	1,663,834	149	1033	ST-1034	ERP000129	ERR024466
128	goose137	UK	2007	goose	1,600,143	53	137	ST-45	ERP000129	ERR024468
129	goose696	UK	2007	goose	1,561,449	143	696	ST-1332	ERP000129	ERR024469
130	duck702	UK	2007	duck	1,669,953	108	702	ST-702	ERP000129	ERR024470
131	duck45	UK	2007	duck	1,616,162	60	45	ST-45	ERP000129	ERR024462
